# When more is more: redundant modifiers can facilitate visual search

**DOI:** 10.1186/s41235-021-00275-4

**Published:** 2021-02-17

**Authors:** Gwendolyn Rehrig, Reese A. Cullimore, John M. Henderson, Fernanda Ferreira

**Affiliations:** 1 Department of Psychology, University of California, One Shields Ave, Davis, CA 95616-5270 USA; 2 Center for Mind and Brain, University of California, One Shields Ave, Davis, CA 95616-5270 USA

**Keywords:** Gricean maxims, Visual search, Overinformativity, Adjectives, Template guidance

## Abstract

**Abstract:**

According to the Gricean Maxim of Quantity, speakers provide the amount of information listeners require to correctly interpret an utterance, and no more (Grice in Logic and conversation, 1975). However, speakers do tend to violate the Maxim of Quantity often, especially when the redundant information improves reference precision (Degen et al. in Psychol Rev 127(4):591–621, 2020). Redundant (non-contrastive) information may facilitate real-world search if it narrows the spatial scope under consideration, or improves target template specificity. The current study investigated whether non-contrastive modifiers that improve reference precision facilitate visual search in real-world scenes. In two visual search experiments, we compared search performance when perceptually relevant, but non-contrastive modifiers were included in the search instruction. Participants (*N*_Exp. 1_ = 48, *N*_Exp. 2_ = 48) searched for a unique target object following a search instruction that contained either no modifier, a location modifier (Experiment 1: *on the top left*, Experiment 2: *on the shelf*), or a color modifier (*the black lamp*). In Experiment 1 only, the target was located faster when the verbal instruction included either modifier, and there was an overall benefit of color modifiers in a combined analysis for scenes and conditions common to both experiments. The results suggest that violations of the Maxim of Quantity can facilitate search when the violations include task-relevant information that either augments the target template or constrains the search space, and when at least one modifier provides a highly reliable cue. Consistent with Degen et al. (2020), we conclude that listeners benefit from non-contrastive information that improves reference precision, and engage in rational reference comprehension.

**Significance statement:**

This study investigated whether providing more information than someone needs to find an object in a photograph helps them to find that object more easily, even though it means they need to interpret a more complicated sentence. Before searching a scene, participants were either given information about where the object would be located in the scene, what color the object was, or were only told what object to search for. The results showed that providing additional information helped participants locate an object in an image more easily only when at least one piece of information communicated what part of the scene the object was in, which suggests that more information can be beneficial as long as that information is specific and helps the recipient achieve a goal. We conclude that people will pay attention to redundant information when it supports their task. In practice, our results suggest that instructions in other contexts (e.g., real-world navigation, using a smartphone app, prescription instructions, etc.) can benefit from the inclusion of what appears to be redundant information.

## Introduction

Suppose you and several friends get together for a picnic in the park. When your friends realize they left a blanket in the car, you are asked to retrieve the blanket while they scout out a good picnic spot. Your friend hands you a set of car keys and says “it’s the green Mazda” as you make your way to the parking lot. In this context, the speaker—your friend—knows that you are searching for a car among other cars, and provided extra information about the target car (make and color) to help you find it. If your friend’s car is the only car in the parking lot when you arrive, however, the description would be overinformative, and therefore suboptimal from an audience design perspective, which assumes that linguistic expressions should be included only as required to avoid referential ambiguity (Grice 1975). The current study investigates whether overinformative modifiers—those that add information about unique targets in a scene beyond what is minimally required for identification—facilitate visual search, despite adding redundancy to an utterance.

According to audience design theories of communication (Grice 1975; Clark and Murphy 1982; Konopka and Brown-Schmidt 2014), speakers craft utterances with the listener in mind such that they account for common ground between speaker and listener (Clark and Murphy 1982), provide relevant context, and are efficient (Gibson et al. 2019). Grice (1975) famously dubbed the tendency for speakers and listeners to accommodate each other’s communicative needs the “Cooperative Principle” of conversation, and outlined Maxims (best practices) that speakers follow in cooperation with the listener to optimize conversation. In the current study, we focus on the Maxim of Quantity, which states that speakers should provide enough information for listeners to correctly identify the intended referent, and no more (Grice 1975).

Gricean Maxims are guidelines for communication, not inviolable rules that speakers obey strictly. Indeed, speakers systematically violate the Maxim of Quantity in particular (Pechmann 1989; Belke and Meyer 2002; Sedivy 2003; Gatt et al. 2011; Koolen et al. 2013; Westerbeek et al. 2015; Rubio-Fernández 2016; Gatt et al. 2017; Degen et al. 2020). Hereafter we will use the term “non-contrastive” to refer to a referential expression that includes modifiers which are not strictly required for unique identification (e.g., “the red pen” in a context with only a single pen). Overinformative referring expressions frequently include non-contrastive color descriptors (Pechmann 1989; Belke and Meyer 2002; Sedivy 2003; Gatt et al. 2011; Koolen et al. 2013; Engelhardt and Ferreira 2016; Degen et al. 2020), except when the color is highly typical of the object (e.g., “the yellow banana”; Westerbeek et al. 2015; Rubio-Fernández 2016). Speakers are more likely to produce overinformative referring expressions that describe atypical object properties (e.g., “the brown banana”) than highly typical object features (Sedivy 2003; Mitchell et al. 2013; Westerbeek et al. 2015; Degen et al. 2020), and are more likely to include color modifiers than size modifiers (Sedivy 2003). The tendency for speakers to overinform increases with stimulus complexity (Koolen et al. 2013; Davies and Katsos 2013; Gatt et al. 2017; Degen et al. 2020). In a series of production experiments, Degen et al. (2020) replicated these patterns: Speakers systematically included non-contrastive information in referring expressions, especially when describing complex stimuli and atypical object properties. The authors fit a Rational Speech Act model with continuous semantics to the data and found speakers elected to include non-contrastive modifiers when those modifiers made the reference more precise, and presumably more useful to the interlocutor. In sum, speakers include strictly non-contrastive information strategically, resulting in referring expressions that are appropriately informative because the non-contrastive information is still useful.

Under a classical interpretation of Gricean Maxims, it should be more difficult for an interlocutor to arrive at the correct reference interpretation if the referential expression is either over- or underinformative. This prediction follows from the Gricean idea that listeners assume speakers are economical in their use of linguistic expressions, and therefore will use a modifier to infer the existence of a set of items denoted by the head noun rather than a single item. If only one item in fact is present, listeners will be momentarily confused. However, there is mixed evidence regarding how violations of the Maxim of Quantity affect an interlocutor’s interpretation of the reference (Engelhardt et al. 2006, 2011; Arts et al. 2011; Davies and Katsos 2013; Engelhardt and Ferreira 2016; Toutouri et al. 2017). For example, Visual World Paradigm tasks—which employ displays that are similar to visual search arrays—have shown that listeners experience comprehension difficulty when interpreting an overinformative description (Engelhardt et al. 2006) and further exhibit processing difficulties for both under- and overinformative utterances (Davies and Katsos 2013). In an attentional-cueing task, Engelhardt et al. (2011) found longer response times and an N400 following overinformative modifiers, indicating a processing penalty associated with unexpected redundant information. It is important to note that overmodification may have been detrimental in the cases discussed above because the non-contrastive modifiers did not improve reference precision (Degen et al. 2020). Other studies have reported *facilitation* of reference interpretation when the referring expression contains redundant modifiers. Arts et al. (2011) found violations of the Maxim of Quantity facilitated target object identification among an array of objects when the overinformative modifiers communicated perceptually relevant information (size, color, shape), or spatial information about the target’s location in the array (e.g., left). Toutouri et al. (2017) found overinformative modifiers facilitated search for a target in an object array when the modifier reduced reference entropy: For example, “blue” was helpful when there were few blue objects, and not when many objects were blue. In other words, there was a benefit of non-contrastive modifiers when those modifiers made the referring expression more precise, consistent with Degen et al. (2020). To summarize, non-contrastive modifiers appear to impede reference interpretation when they add noise (e.g., when stimuli are simple or the “speaker” is not reliable), but may facilitate reference interpretation when they improve reference precision.

The predictions about reference interpretation that follow from the classical Gricean perspective—and have been partially supported empirically in psycholinguistic work (Engelhardt et al. 2006, 2011; Davies and Katsos 2013)—are rather counterintuitive in the context of visual search. In the hypothetical car search scenario, the expression “it’s the green Mazda” is overinformative because the color and make are non-contrastive, and so the expression arguably violates the Gricean Maxim of Quantity. However, the overinformative details (the make and color of the car) are perceptually relevant and therefore clearly useful for visual search. Well-defined target information has been shown to facilitate template-based guidance of search for a target object in real-world scenes (Vickery et al. 2005; Malcolm and Henderson 2009, 2010; Castelhano and Heaven 2010; Reeder and Peelen 2013; Bahle et al. 2019). In template-based guidance of visual search, the observer uses a target object cue (e.g., a word or picture) to form a template of the target object in visual working memory, which is subsequently compared to the scene during search (Rao et al. 2002; Schmidt and Zelinsky 2009; Malcolm and Henderson 2009). Targets are located faster when the template is more specific (e.g., a picture of the target vs. the name of the target object; Malcolm and Henderson 2009, 2010; Castelhano and Heaven 2010; Schmidt and Zelinsky 2009; Bravo and Farid 2009), and when the target is a highly typical exemplar of the object category (Castelhano et al. 2008; Maxfield et al. 2014)—though typicality only reduced the time required to verify the target after it was initially fixated (Castelhano et al. 2008). Counter to the Gricean prediction, there is an additive benefit when multiple cues are provided (Malcolm and Henderson 2010; Castelhano and Heaven 2010; Hout and Goldinger 2015). However, the degree to which additional information is beneficial depends on how consistent features are within the object category (e.g., how noisy object feature cues are; Hout et al. 2017), which is consistent with Degen et al. (2020). Target templates held in working memory can incorporate the shape of the target objects as well as diagnostic object parts (e.g., a wheel on a car; Reeder and Peelen 2013), and can incorporate color information (Bahle et al. 2019), evidenced through attention capture by distractor objects with the same shape (Reeder and Peelen 2013) or color (Bahle et al. 2019) as the template. Note that when there were only two target object categories, a single letter (the first letter of the target category name) was sufficient to build object shape information into the target object template (Reeder and Peelen 2013). Based on these findings, we would expect non-contrastive descriptors to facilitate visual search so long as they enrich the target search template.

It is unclear whether the mixed evidence on how violations to the Maxim of Quantity influence reference processing in the psycholinguistic literature, and the mismatch between the reference processing literature and empirical work on templated-based visual search, is due to differences in the paradigms used in each field. For example, reference processing experiments tend not to use complex real-world scenes, and often involve button press or typed responses. When eye-movements are recorded, they are not typically analyzed in the same way in the psycholinguistic literature as in the visual search literature; in Visual World Paradigm tasks, fixations made to each image in a search array during a target period of the auditory stimulus are aggregated (e.g., averaged across trials) for analysis. Furthermore, the literature on template-guided visual search has shown that some information improves target template—and template-guided search by association—more than others, and in many of these paradigms the information is provided in written form. It is possible that theories of reference processing can speak to why certain types of information, presented in linguistic and acoustic form, are more useful than others. The discrepancy provides a fruitful opportunity for cross-disciplinary research. In the current study, we investigated whether the beneficial effects of non-contrastive modifiers on reference processing (Arts et al. 2011; Toutouri et al. 2017) generalize to visual search in real-world scenes, an understudied topic in both psycholinguistics and visual cognition. Real-world scenes benefit from rapid scene gist extraction (Castelhano and Henderson 2007), are processed more efficiently than cartoons or other simplified displays (Henderson and Ferreira 2004), and better approximate real-world environments. Because real-world scenes are complex stimuli, we expect any non-contrastive modifiers that convey task-relevant information to improve reference precision (following Degen et al. 2020). In two experiments, observers searched real-world scenes for a unique target object. We manipulated reference specificity by modifying the search instruction to add either a perceptually relevant target feature or information about the target location.

Based on evidence that target object templates can contain color information (Bahle et al. 2019), in both experiments we added non-contrastive but perceptually relevant information using a color modifier (e.g., *Find the black lamp*). Following Arts et al. (2011), in Experiment 1 we added target location information using a prepositional phrase after the target object’s name that specified which screen quadrant the target was located in (e.g., *Find the lamp on the top left*). In Experiment 2, we instead expressed location information relative to an anchor object in the scene (Boettcher et al. 2018). Anchor objects are typically larger objects (e.g., a desk, table, bookshelf, etc.) on which target objects are likely to be located (e.g., *Find the lamp on the shelf*). In both experiments, performance on trials in which the search instruction included non-contrastive modifiers was compared to trials in which only the target object was mentioned in the search instruction (e.g., *Find the lamp*).

**Fig. 1:**
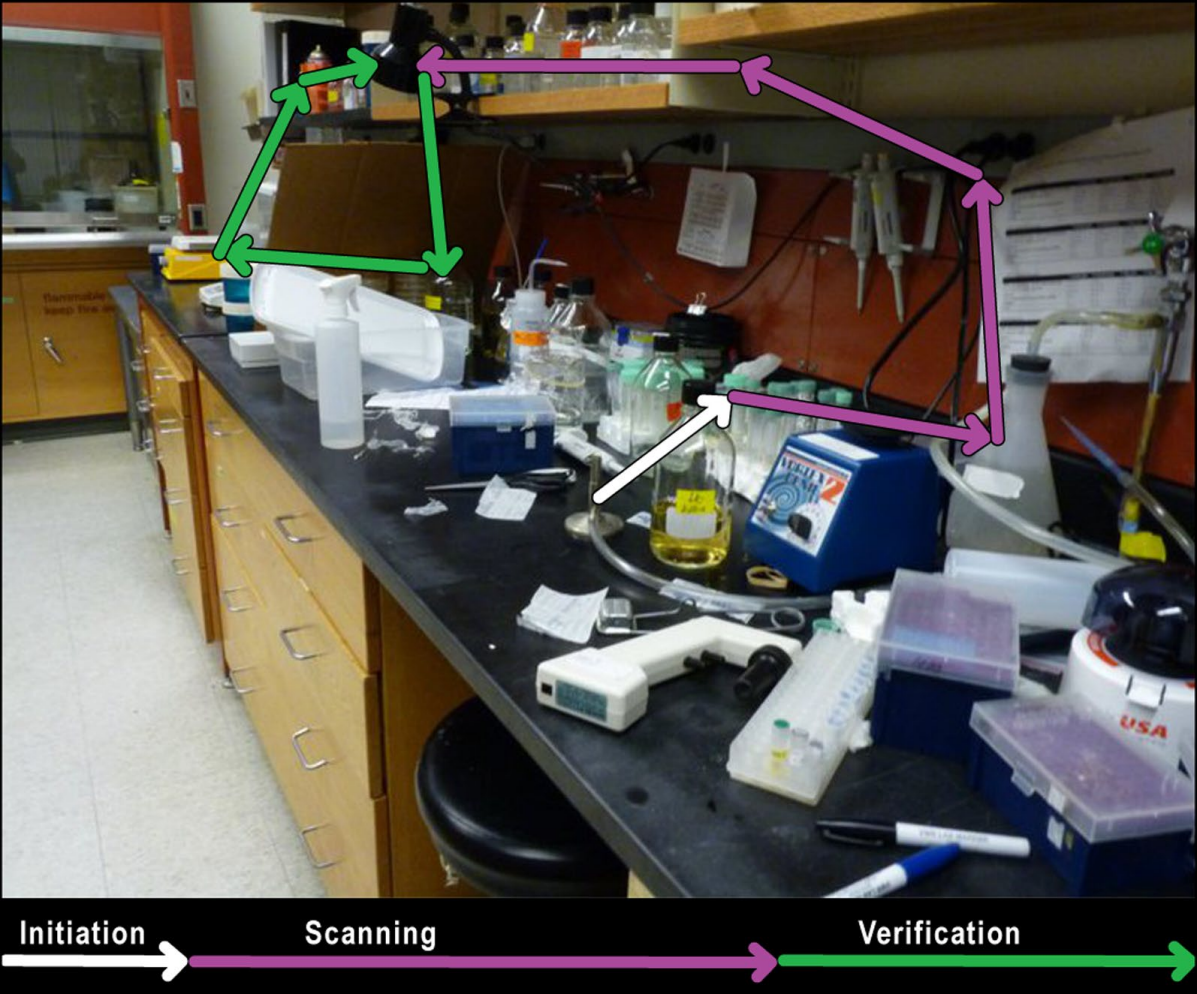
An example scan path divided into search epochs. In this scene, the target is the black lamp clamped to a shelf. Initiation (white) is when the observer begins to search the scene, defined as the time between stimulus onset and the first saccade. Scanning (purple) is the search epoch, defined as the time between the first saccade and the first fixation on the target object. Verification (green) is when the observer confirms that the object they fixated is the target object, operationalized as the time between initially fixating the target object and the subject’s response. Scene image courtesy of Imgur

Because Malcolm and Henderson (2009, 2010) found a target template advantage specifically when observers scanned the scene (scanning time) and in the time interval between finding the target and responding accordingly (verification time), we similarly divided the trial period into the same three discrete search epochs to determine which search epoch may benefit from the redundant modifiers chosen (Fig. 1). Initiation time was the latency of the first saccade following scene onset, at which point the eye first moved to search the scene (Fig. 1, white arrow). Malcolm and Henderson (2009, 2010) did not find a benefit of target template specificity on initiation times; therefore, we do not expect this measure to be sensitive to reference precision. Scanning time was the primary search epoch, defined as the time taken to fixate on the target object after the first saccade (Fig. 1, purple arrows). Verification time was the time to confirm the fixated object was indeed the target, defined as the time between the first fixation on the target object and the subject’s response (Fig. 1, green arrows). Reaction time, a common measure of search efficacy, was defined as the time between the start of the trial and the subject’s response.

We hypothesized that a non-contrastive color modifier would augment the target search template and facilitate target template-guided search relative to when no modifier is present (Bahle et al. 2019). We also hypothesized that location modifiers would facilitate visual search by constraining the region of the display to be searched (Arts et al. 2011). Based on the finding that non-contrastive modifiers improve reference precision and are therefore useful for visual search (Degen et al. 2020), then following Malcolm and Henderson (2009), we predicted non-contrastive, redundant modifiers to facilitate visual search. Specifically, in both experiments we predicted that the duration of the primary search epoch (scanning time) and confirmation epoch (verification time) would be shorter when modifiers appeared in the search instruction in the presence of non-contrastive modifiers in both experiments.^1^

## Experiment 1

In Experiment 1, we compared search performance when the search instruction included non-contrastive, redundant modifiers to performance when no such modifiers were provided. Specifically, we included either the color of the target object (e.g., *Find the black lamp*) to augment the target object template, the location of the target object in the scene (e.g., *Find the lamp on the upper left*) to constrain the region of the scene to be searched, or no additional information (e.g., *Find the lamp*). We predict that redundant, non-contrastive modifiers that constrain the relevant object colors and locations within a scene will facilitate visual search. We expect scanning and verification times to be faster when there is a redundant modifier in the referring expression.

## Experiment 1: Method

### Stimulus selection

Forty-two scene candidates were selected from Google image search. All scenes depicted human-made environments (e.g., kitchens, offices, drawers) and each contained only one instance of the target object type (e.g., only one mug).

Prior to the eye-tracking study, we conducted a norming study to verify that the intended target in each scene was relatively easy to find and that its color was easily identifiable. Fourteen native English speaking undergraduates enrolled at UC Davis completed a Qualtrics survey. Each of the 42 scenes was presented individually. For each scene, subjects were instructed to report separately the location of the target object and its color. Responses were recorded via text box. Prior to the 42 experimental trials, subjects viewed an example trial in which a scene was displayed along with its location relative to another object in the scene (e.g., *on the desk*) and its color (e.g., *white*).

Results of the norming study were used to exclude scenes as follows. Two scenes were excluded because subjects spent over 30 s searching for the object or failed to locate the object. An additional two scenes were excluded because subjects reported that more than one instance of the object was present in the scene. Finally, two more scenes were excluded because subjects did not agree on the identity of the object (e.g., they mistook another object for the target) and because no single color constituted a majority of the color responses. The remaining 36 scenes were presented as stimuli in the eye-tracking experiment.

### Stimulus preparation

For each scene, we defined a rectangular interest area surrounding the target object. The region of interest (ROI) was used to determine when subjects fixated on the target, and to exclude trials in which observers did not fixate the target from analysis.

### Participants

Participants were 50 native English speaking adults enrolled at UC Davis. All subjects had normal or corrected-to-normal visual acuity and normal color vision. Subjects were naive to the purpose of the experiment and provided informed consent to participate. Two of the subjects could not be accurately eye-tracked. Data from the remaining 48 subjects were analyzed.

### Apparatus

The experiment was conducted using an EyeLink 1000 + system with a tower mount. Subjects sat approximately 83 cm from the display monitor. Head movements were stabilized using a chin and forehead rest. Stimuli were displayed at 1024 × 768 pixels in resolution on a 21″ CRT monitor and subtended approximately 36° × 27° visual angle. Viewing was binocular, but eye movements were recorded from the right eye only. Experiment presentation was controlled using SR Research Experiment Builder software.

### Design

The modifier manipulation was implemented via the search instruction subjects received prior to seeing the scene, which was presented in written form in the first display of each trial. The instruction either did not include a modifier (e.g., *Find the lamp*), included a color modifier (*Find the black lamp*), or included a location modifier (*Find the lamp on the upper left*). The color modifier was chosen from the majority response provided in the norming study, and the location modifier was chosen by determining which scene quadrant (upper left, lower left, upper right, lower right) contained the target object.

Each experimental session consisted of 36 experimental trials. The modifier manipulation was implemented within-subject such that 12 trials did not include a modifier, 12 trials included a color modifier, and the other 12 included a location modifier. The scenes and all modifiers were counterbalanced and equally distributed across three lists. Subjects were assigned to one of the three lists at random.

### Procedure

Subjects were first instructed to search for targets in each scene and to press a button on the button box upon locating the target object. Prior to the experimental trials, a calibration procedure was performed to map eye position to screen coordinates. Calibration was successful if the average error fell below 0.49° and maximum error was below 0.99°. Fixations and saccades were parsed with EyeLink’s standard algorithm using velocity and acceleration thresholds (30°/s and 9500°/s^2^; SR Research 2017a).

Calibration was maintained throughout the experiment using a drift correction procedure to check and correct for calibration drift. Prior to each trial, a central fixation cross was presented on screen, and the experimenter pressed a button to continue unless the drift check error exceeded 0.99° visual angle, in which case the experimenter repeated the calibration procedure.

**Fig. 2:**
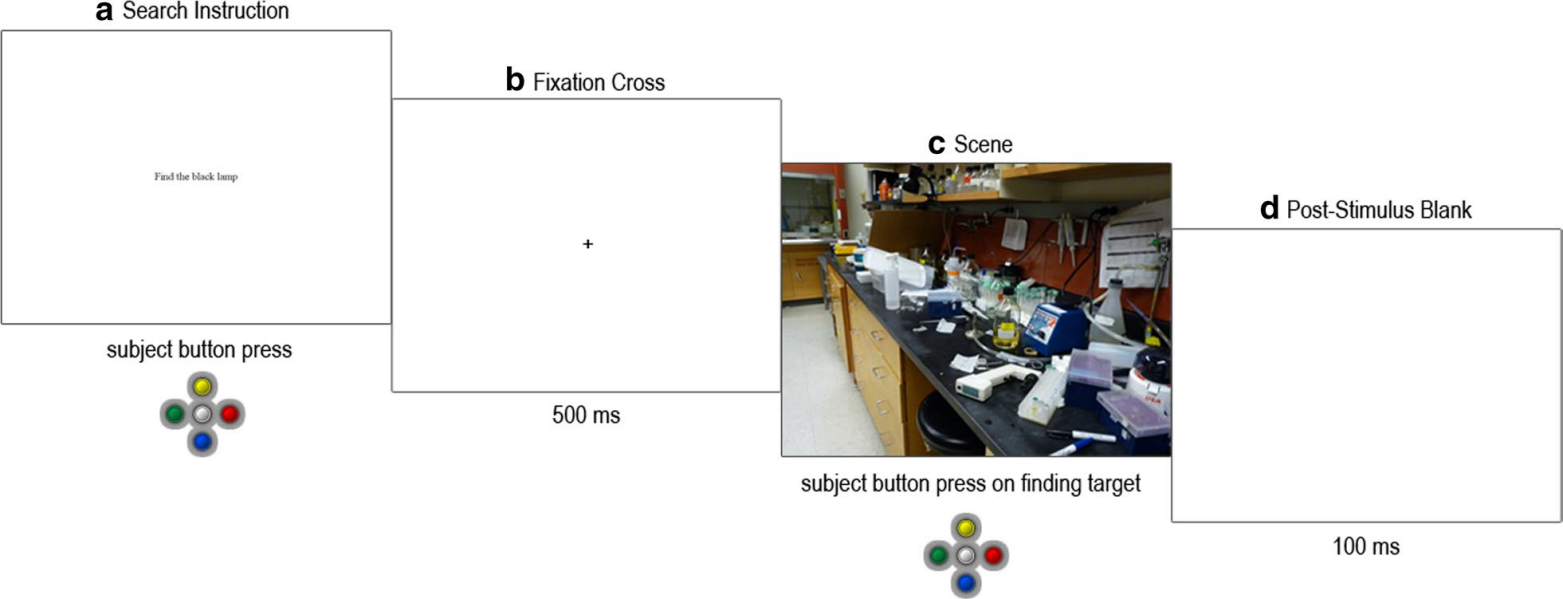
Trial procedure schematic. **a** The search instruction (e.g., *Find the black lamp*) was presented in the center of the screen until the subject pressed a button to proceed. **b** A central fixation cross was presented for 500 ms. **c** The scene was presented until the subject pressed a button to indicate that the target object had been found. **d** A blank screen was presented for 100 ms, after which the trial procedure repeated

Successful initial calibration was followed by 3 practice trials. A trial proceeded as follows (Fig. 2). After the drift check procedure, the search instruction (e.g., *Find the black lamp*) was presented in black 20 pt Times New Roman font on the center of a white screen. The instruction persisted until the subject pressed a button, after which a central fixation cross appeared for 500 ms, followed by the scene. The scene persisted until the subject pressed a button upon locating the target, at which point response time was recorded. After a 100-ms blank screen, the next trial began.

The procedure for the experimental trials and the practice trials was identical. After completing three practice trials, subjects completed 36 experimental trials. Trial presentation was randomized without replacement.

### Data treatment

Prior to analysis, data were inspected in Data Viewer (SR Research 2017b) by the second author. Trials were excluded from analysis entirely (1) if a fixation landed within the target ROI immediately after the fixation cross (61 trials in the location modifier condition only), (2) if no fixations landed in or near the target ROI, in which case observers may have failed to find the target or may have mistaken another object for the target, and (3) trial duration outliers that were over 3 standard deviations above the mean trial duration. We excluded 278 trials (out of 1728 trials total) from analysis using these criteria: 66 in the control condition (3.8% of all trials), 65 in the color modifier condition (3.8% of all trials), and 147 in the location modifier condition (8.5% of all trials). Data from the remaining 1450 trials were analyzed.

### Measures

We measured reaction time to gauge overall search performance, defined as the time in milliseconds between the start of a trial and the subject’s response, which terminated the trial. Following Malcolm and Henderson (2009), we divided each trial into three search epochs: initiation, scanning, and verification. Initiation time was equivalent to initial saccade latency, the milliseconds that elapsed between scene onset and when the eye first moved to search the scene. Short initiation latencies (≤ 90 ms) were excluded from analysis. Scanning time was the time taken to traverse the scene before finding the target, defined as the time between initiation (initial saccade latency) and when the target object was first fixated. Verification time was defined as the time, in milliseconds, between when the observer first fixated the target object and the end of the trial.

### Analysis

Each of the dependent measures (initiation time, scanning time, verification time, and reaction time) was analyzed in turn using a Bayesian mixed-effects model implemented using the brms package in R (Bürkner 2017, 2018). To facilitate model convergence, the adapt_delta parameter was set to 0.999999999999 for each model, and max_treedepth was set to 15. Because the measures analyzed were ex-Gaussian distributed, each model used an ex-Gaussian linking function. Unless otherwise noted, each model used the default (weakly informative) priors and was maximally specified, with modifier condition as a fixed effect, and random effects of item (scene) and subject with uncorrelated random intercepts and slopes, and all other parameters (e.g., number of iterations) were set to the default. The modifier condition variable was centered prior to analysis, and the reference level was always the no modifier condition (e.g., *Find the lamp*). We consider differences to be reliable if the 95% credible interval (reported as an equal-tail interval) for the comparison does not contain zero, in which case the true value of *β* is unlikely to be zero (Nicenboim and Vasishth 2016).

## Experiment 1: Results

We predicted that the presence of a redundant, non-contrastive modifier would facilitate search, despite ostensibly violating the Gricean Maxim of Quantity. We predicted that color and location modifiers would reduce all search epoch durations relative to the no modifier control.

### Initiation time

**Fig. 3:**
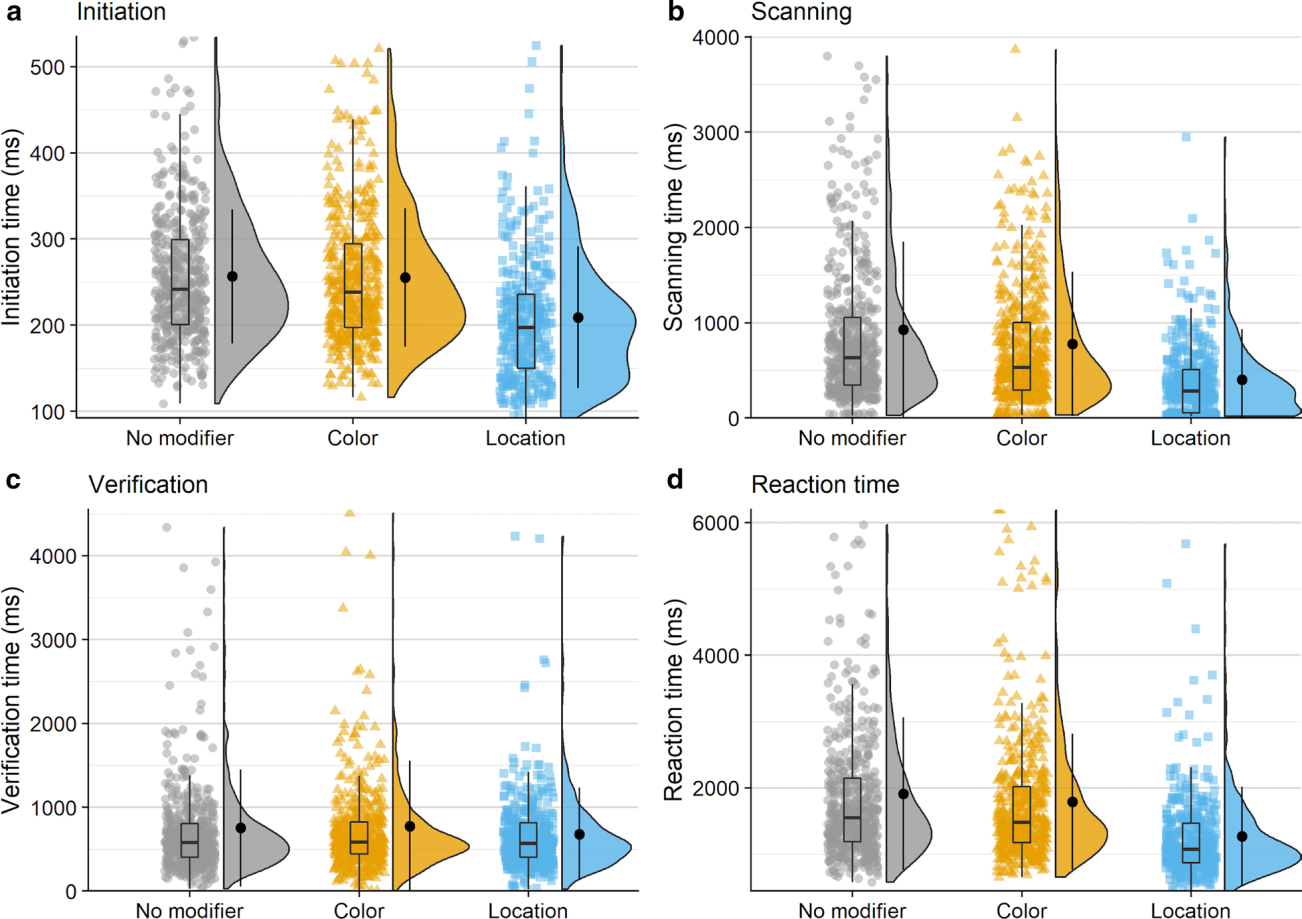
Raincloud plots depicting data for Experiment 1, oriented vertically for legibility. Points on the left side of each raincloud show raw data. Box plots, superimposed over the raw data, indicate the median value (middle horizontal line), the first and third quartiles (top and bottom lines in box), and 1.5 × the interquartile range: the distance between the first and third quartiles (whiskers). Violin plots show the range (minimum and maximum) and density (width) of the data. Isolated points on the right side of each raincloud indicate the mean, and error bars on said points reflect one standard deviation. For ease of visualization, the bottom 99% of the data are shown

On average, observers made an initial saccade 243 ms after scene onset (*M* = 243, SD = 83). Initiation time was longest on average in the no modifier condition (*M* = 257 ms, SD = 78 ms), followed by the color modifier condition (*M* = 255 ms, SD = 81 ms), and was fastest when a location modifier was present (*M* = 209 ms, SD = 82 ms; see Fig. 3a). Overall, this trend is not numerically consistent with our predictions because initiation times differed across modifier conditions.

**Fig. 4:**
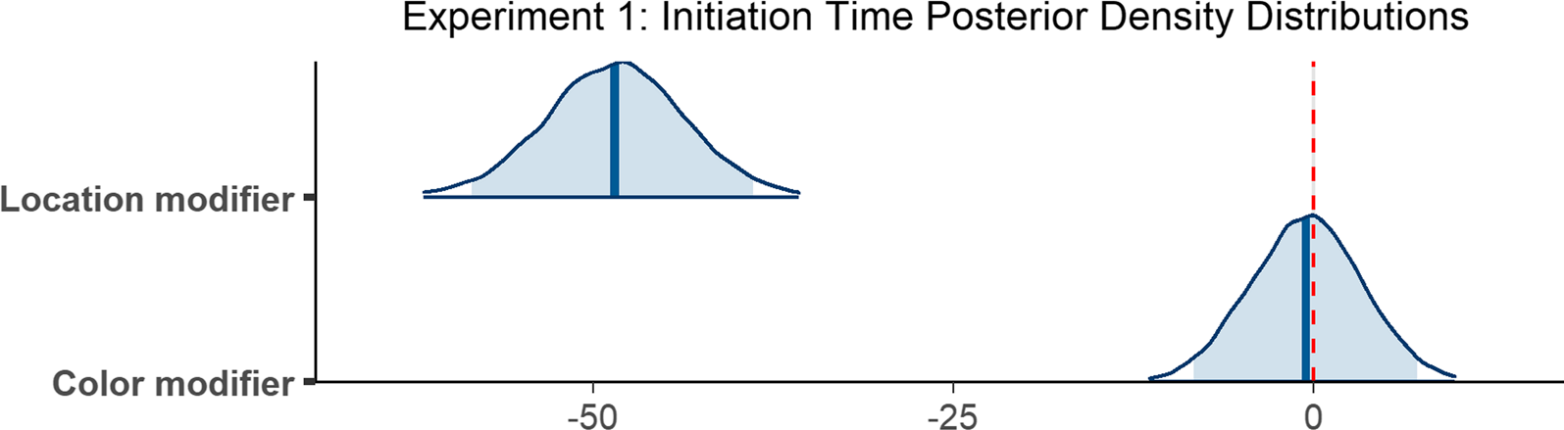
Posterior density distributions for initiation time *β* parameter estimates for each level of the fixed effect relative to the reference level. Light blue shaded regions indicate the 95% credible interval and blue vertical lines indicate the mean of the distribution. The red vertical dashed line highlights 0 on the *x* axis. If the red line falls within the light blue shaded region, the estimate is not reliably different from zero and interpreted as a null result

The model ran for 8000 iterations. Analysis using this model revealed no reliable difference between the color modifier and no modifier conditions (*β* = − 0.50, 95% CI =  [− 8.33 7.20]). Initiation times in the location modifier condition, however, did differ reliably from the no modifier condition (*β* = − 48.54, 95% CI = [− 58.48 − 38.92]; see Fig. 4 for posterior draw visualizations).

### Scanning time

Observers required 720 ms on average to scan the scene between executing an initial saccade and fixating the target for the first time (*M* = 720 ms, SD = 795 ms). The scanning epoch was longest when no modifier was provided (*M* = 928 ms, SD = 924 ms), shorter in the presence of a color modifier (*M* = 779 ms, SD = 759 ms), and shortest when observers were given a location modifier (*M* = 402 ms, SD = 531 ms; Fig. 3b). The decrease in mean scanning time in the presence of a non-contrastive modifier is consistent with our predictions.

**Fig. 5:**
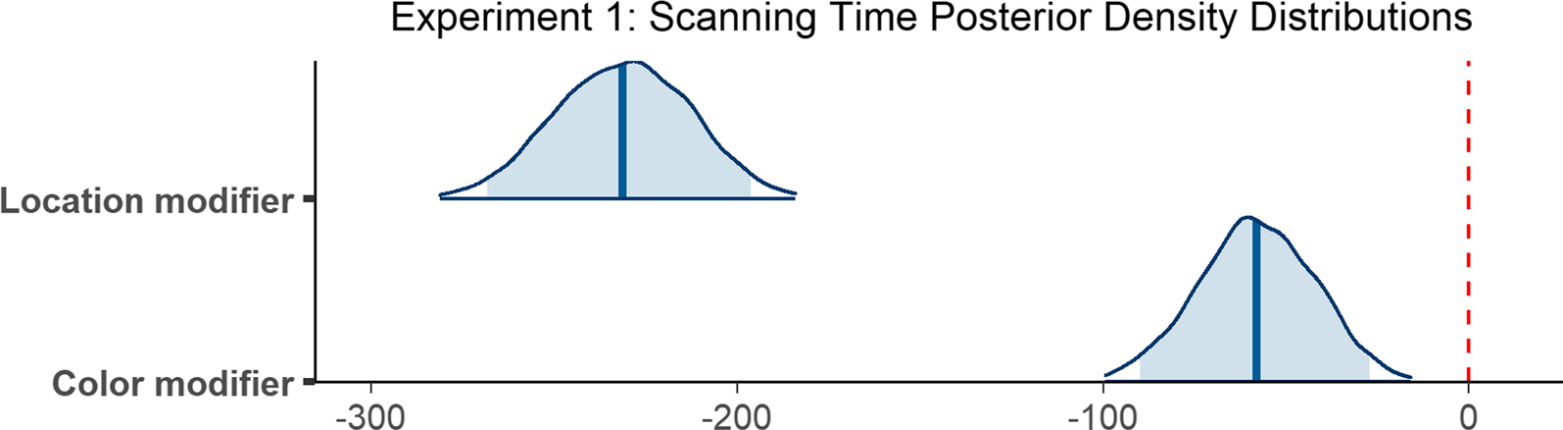
Posterior density distributions for scanning time *β* parameter estimates for each level of the fixed effect relative to the reference level. Light blue shaded regions indicate the 95% credible interval and blue vertical lines indicate the mean of the distribution. The red vertical dashed line highlights 0 on the *x* axis. If the red line falls within the light blue shaded region, the estimate is not reliably different from zero and interpreted as a null result

The final model included random slopes for condition in the subject random effect, and random intercepts in the item random effect. The model did not converge with weakly informative priors, but did converge using a more informative prior with a Student *t* distribution on *β* parameter estimates (*df* = 3, *μ* = 0, *σ* = 20) and on standard deviations for the by-subject random slopes (*df* = 3, *μ* = 0, *σ* = 10), and an exponential prior (*μ* = 25) on the standard deviations corresponding to the location modifier condition for the by-subject random slopes. According to the final model, the difference in scanning time when a color modifier was provided as opposed to no modifier at all was reliable (*β* = − 58.01, 95% CI = [− 89.92 − 27.11]), as was the difference in time when a location modifier was provided compared to no modifier (*β* = − 231.42, 95% CI = [− 268.41 − 196.21]; Fig. 5).

### Verification time

The time between initially fixating the target and trial termination, or verification time, was 740 ms on average (*M* = 740 ms, SD = 693 ms). Verification took longest when a color modifier was present (*M* = 776 ms, SD = 780 ms), was slightly less long when no modifier was present (*M* = 752 ms, SD = 701 ms), and was fastest when a location modifier was provided *(M* = 681 ms, SD = 556 ms; Fig. 3c).

**Fig. 6:**
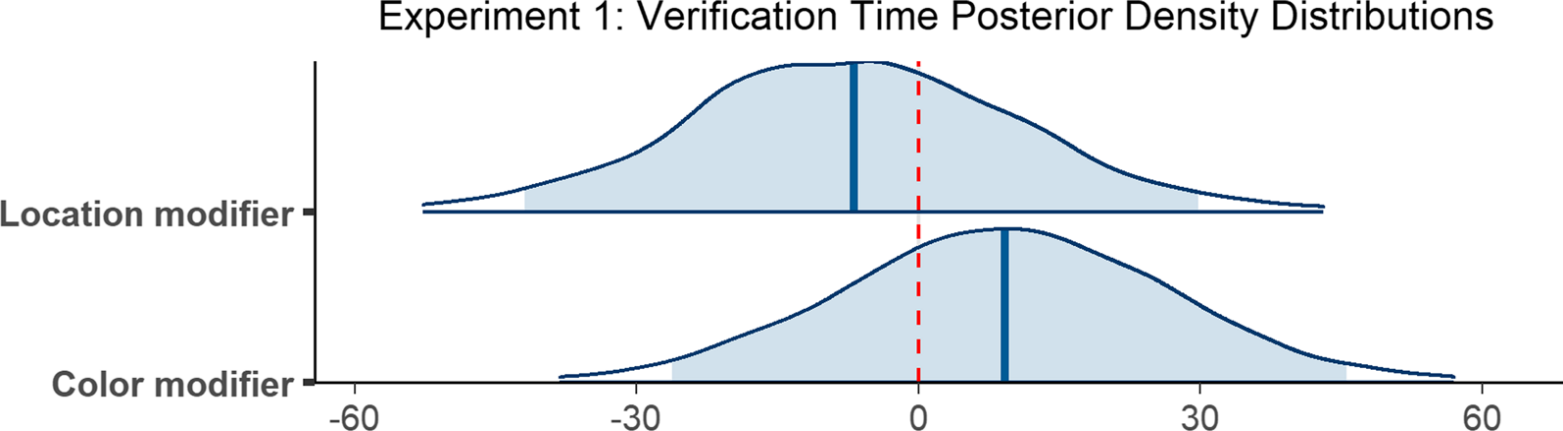
Posterior density distributions for verification time *β* parameter estimates for each level of the fixed effect relative to the reference level. Light blue shaded regions indicate the 95% credible interval and blue vertical lines indicate the mean of the distribution. The red vertical dashed line highlights 0 on the *x* axis. If the red line falls within the light blue shaded region, the estimate is not reliably different from zero and interpreted as a null result

The model revealed that none of the differences reported above were reliable. Verification time did not differ reliably when a color modifier was present compared to when no modifier was used (*β* = 9.22, 95% CI = [− 26.23 45.58]), and the presence of a location modifier similarly did not yield a reliable difference (*β* = − 6.82, 95% CI = [− 41.96 29.73]; Fig. 6).

### Reaction time

On average, observers took 1677 ms to indicate that they had successfully found the target object (*M* = 1677 ms, SD = 1038 ms). Reaction time was longest when no modifier was present (*M* = 1910 ms, SD = 1152 ms), was shorter when a color modifier was present (*M* = 1790 ms, SD = 1026 ms), and shortest when a location modifier was used (*M* = 1265 ms, SD = 751 ms; Fig. 3d).

**Fig. 7:**
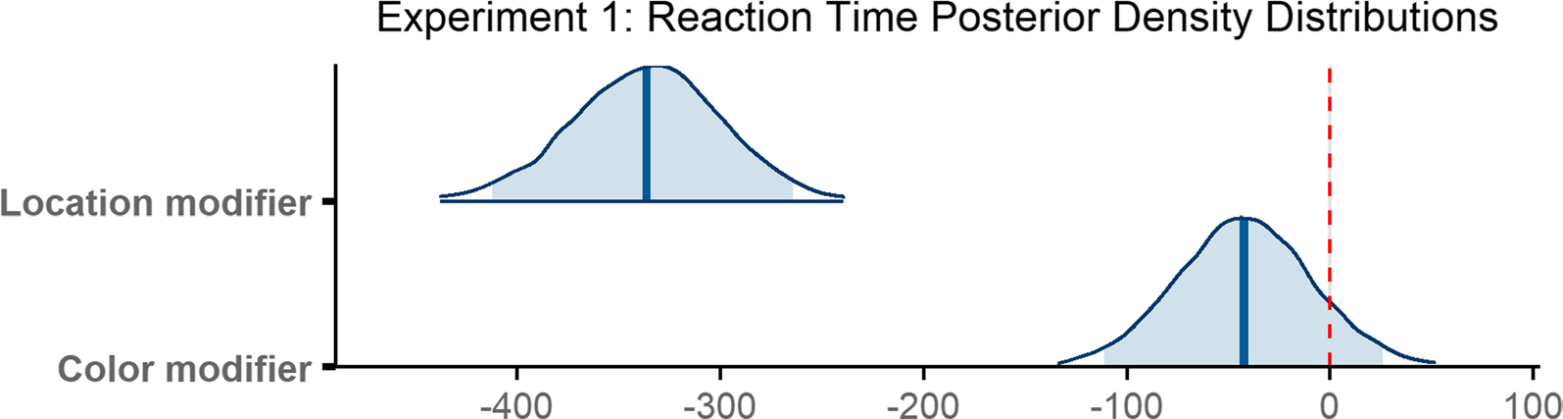
Posterior density distributions for reaction time *β* parameter estimates for each level of the fixed effect relative to the reference level. Light blue shaded regions indicate the 95% credible interval, and blue vertical lines indicate the mean of the distribution. The red vertical dashed line highlights 0 on the *x* axis. If the red line falls within the light blue shaded region, the estimate is not reliably different from zero and interpreted as a null result

The model did not converge with weakly informative priors, but did converge using more informative priors with a Student t distribution on *β* parameter estimates (*df* = 3, *μ* = 0, *σ* = 150), on standard deviations for random slopes (*df* = 3, *μ* = 0, *σ* = 15), and on the *β* parameter of the exponential distribution (*df* = 3, *μ* = 0, *σ* = 25), and an exponential prior on the standard deviations (*μ* = 50) corresponding to the by-item random intercepts and to the no modifier condition for the by-subject random slopes. The final model ran for 6000 iterations. According to the model, reaction time did not reliably differ when a color modifier was provided as opposed to no modifier at all (*β* = − 42.36, 95% CI = [− 110.98 25.97]), but did differ reliably when a location modifier was provided compared to no modifier (*β* = − 336.49, 95% CI = [− 412.26 − 264.52]; Fig. 7).

To summarize, initiation times were reliably faster when a location modifier was present, and did not differ when a color modifier was present as opposed to no modifier at all. Both color modifiers and location modifiers facilitated scanning times, which resulted in shorter scanning epoch durations. There was no difference between any of the modifier conditions with respect to the verification epoch. Only the location modifier condition facilitated reaction times.

## Experiment 1: Discussion

We predicted that redundant, non-contrastive modifiers that constrain the target object colors and locations within a scene would facilitate visual search. Specifically, we expected search to be faster when a modifier was provided. Only scanning time showed the predicted facilitation for search instructions that contained non-contrastive modifiers. Counter to prior findings (Malcolm and Henderson 2009, 2010), we did not find a benefit of modifiers during the verification epoch. Consistent with our predictions, a non-contrastive color modifier reliably facilitated search during the scanning epoch, likely because it augmented the target object template.

The presence of a location modifier facilitated search, resulting in shorter scanning epoch durations, shorter response times, and, surprisingly, shorter initiation times. The location modifier in Experiment 1 was clearly beneficial overall. While the location modifier was not a contrastive modifier with respect to the target object, it was contrastive with respect to the spatial layout of the scene, and because the information it provided was uniquely relevant to visual search, the benefits of narrowing the region of the display to be searched outweighed the costs associated with processing additional linguistic material. We conducted a second experiment to determine whether the observed benefit of location information would hold when the modifier instead expressed the location of the target object relative to another object in the scene.

## Experiment 2

In Experiment 2, we changed the location modifier to express the spatial relationship between target objects and anchor objects in the scene (e.g., *on the desk*; Boettcher et al. 2018) rather than constraining the region of the scene containing the target to a single quadrant. The color modifier was less clearly beneficial in Experiment 1 than the location modifier was. We suspected the color modifier may have been less informative for targets that typically occurred in that color (e.g., *red fire extinguisher*), based on the observation that, in such cases, speakers are less likely to include color adjectives in referring expressions (Sedivy 2003; Mitchell et al. 2013; Westerbeek et al. 2015; Degen et al. 2020). To address this potential limitation, in Experiment 2 we excluded scenes and targets for which the target object’s color was too typical, and added additional scenes.

We again predict non-contrastive modifiers that improve reference precision will facilitate search according to our primary search measures: scanning time, verification time, and reaction time.

## Experiment 2: Method

### Stimulus selection

Fifty-four scene candidates were selected from the stimuli used in Experiment 1, stimuli from a previous study (Henderson and Hayes 2017), the Change Blindness Database (Sareen et al. 2015), and from Google image search. All scenes depicted human-made environments (e.g., kitchens, offices, drawers) and each contained only one instance of the target object type (e.g., only one mug).

As in Experiment 1, we conducted a norming study to verify that the intended target in each scene was relatively easy to find and that its color was easily identifiable. Twenty native English speaking undergraduates enrolled at UC Davis completed a Qualtrics survey. Each of the 54 scenes was presented individually. Like the Experiment 1 norming study, for each scene, subjects were instructed to report separately the location of the target object and its color. Responses were recorded via text box. Additionally, subjects separately rated how typical the target’s color and the location were on a Likert scale (1–7). We added the aforementioned Likert scales to avoid cases where the location or color was highly typical of the object (e.g., fire extinguishers are almost always red), in which case the color modifier used in the search task would be less informative. Prior to the 53 experimental trials, subjects viewed an example trial in which a scene was displayed along with its location (e.g., *on the desk*) and color (e.g., *white*).

In addition to the exclusion criteria used in Experiment 1, we excluded scenes for which color typicality ratings spanned only the highest portion of the Likert scale (rated 6 and 7 only; *n* = 4), scenes for which the location of the target was described as inside another object (a drawer; *n* = 2), scenes for which the color of the object was clearly ambiguous (a solid colored object described with colors from different color families; *n* = 2), one scene that had high variability in both color and location Likert scores (*SD*s > 1.80), and scenes for which the target object was described as multi-colored more than once (*n* = 2). An additional three scenes were excluded because the authors decided they were too sparse to pose a challenge (e.g., search performance in all conditions would likely be at ceiling). In total, we excluded 14 scenes that met our exclusion criteria, and selected the remaining 39 scenes for the second eye-tracking experiment.

### Participants

Participants were 50 native English speaking adults enrolled at UC Davis. All subjects had normal or corrected-to-normal visual acuity and normal color vision. Subjects were naive to the purpose of the experiment and provided informed consent to participate. Two of the subjects could not be accurately tracked. Data from the remaining 48 subjects were analyzed.

### Apparatus

The apparatus was identical to that of Experiment 1. As in Experiment 1, eye movements were recorded from the right eye only, except for two subjects for whom the right eye could not be accurately tracked, and instead eye movements were recorded from the left eye.

### Design

The modifier manipulation was identical to that of Experiment 1, except that the location modifier was a prepositional phrase that identified the location of the target object relative to an anchor object in the scene (*Find the dice on the desk*).

Each experimental session consisted of 39 experimental trials. The modifier manipulation was implemented within-subject such that 13 trials did not include a modifier, 13 trials included a color modifier, and the other 13 included a location modifier. The scenes and all modifiers were equally distributed and counterbalanced across 3 lists. Subjects were assigned to one of the three lists at random.

### Procedure

The experimental procedure for Experiment 2 was identical to that of Experiment 1, except that there were 39 experimental trials.

### Data treatment

We excluded 144 trials corresponding to three scenes that were not equally represented across lists due to a counterbalancing error. An additional 179 trials (out of 1872 trials total) were excluded using the same criteria applied to the data collected in Experiment 1: 61 in the control condition (3.3% of all trials), 55 in the color modifier condition (2.9% of all trials), and 63 in the location modifier condition (3.4% of all trials). Data from the remaining 1549 trials were analyzed.

### Measures

Calculation and definition of dependent measures were identical to that of Experiment 1.

### Analysis

All analyses were carried out using Bayesian mixed-effect models. Model building and analysis criteria were the same as in the data analysis in Experiment 1.

## Experiment 2: Results

Our predictions for Experiment 2 were the same as those for Experiment 1. We expected that non-contrastive modifiers would improve search performance by reducing the duration of the primary search epochs and improving spatial search efficiency.

### Initiation time

**Fig. 8:**
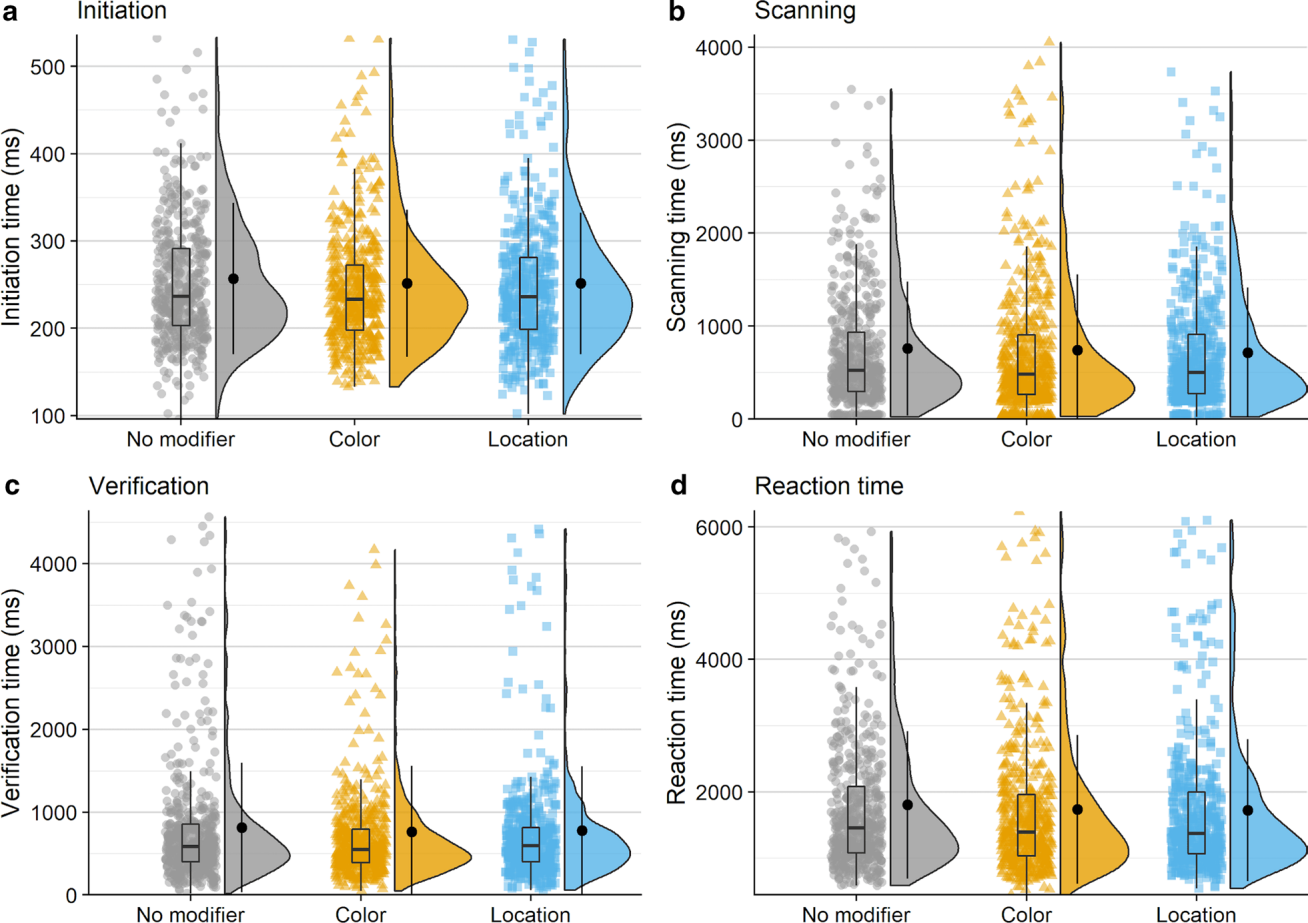
Raincloud plots depicting data for Experiment 2, oriented vertically for legibility. Points on the left side of each raincloud show raw data. Box plots, superimposed over the raw data, indicate the median value (middle horizontal line), the first and third quartiles (top and bottom lines in box), and 1.5 × the interquartile range: the distance between the first and third quartiles (whiskers). Violin plots show the range (minimum and maximum) and density (width) of the data. Isolated points on the right side of each raincloud indicate the mean, and error bars on said points reflect one standard deviation. For ease of visualization, the bottom 99% of the data are shown

Observers made an initial saccade 253 ms after scene onset on average (*M* = 253 ms, SD = 84 ms). Initiation time was longest on average when no modifier was present (*M* = 257 ms, SD = 87 ms), followed by the color modifier condition (*M* = 252 ms, SD = 84 ms), and was fastest when a location modifier was present (*M* = 251 ms, SD = 81 ms; see Fig. 8a).

**Fig. 9:**
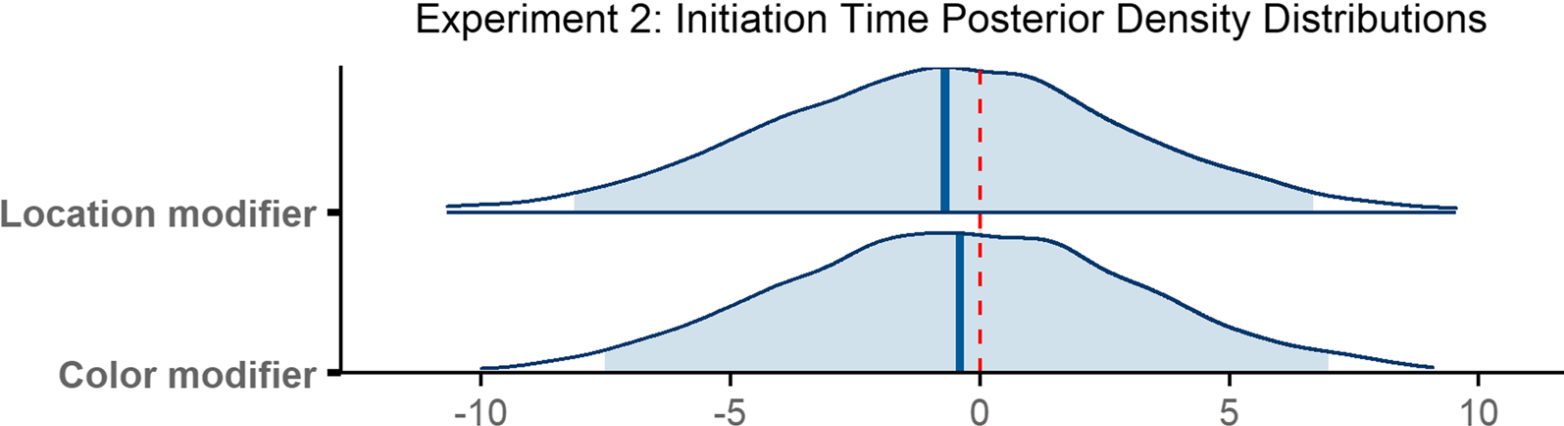
Posterior density distributions for initiation time *β* parameter estimates for each level of the fixed effect relative to the reference level. Light blue shaded regions indicate the 95% credible interval and blue vertical lines indicate the mean of the distribution. The red vertical dashed line highlights 0 on the *x* axis. If the red line falls within the light blue shaded region, the estimate is not reliably different from zero and interpreted as a null result

The final model ran for 4000 iterations. The model revealed no reliable difference in initiation times between the color modifier and no modifier conditions (*β* = − 0.40, 95% CI = [− 7.52 6.99]) or between the location modifier condition and the no modifier condition (*β* = − 0.70, 95% CI = [− 8.14 6.69]; Fig. 9).

### Scanning time

Observers required 741 ms on average to scan the scene between executing an initial saccade and fixating the target for the first time (*M* = 741 ms, SD = 745 ms). The scanning epoch was longest when no modifier was provided (*M* = 759 ms, SD = 721 ms), shorter in the presence of a color modifier (*M* = 745 ms, SD = 811 ms), and shortest when observers were given a location modifier (*M* = 717 ms, SD = 697 ms; see Fig. 7b). The decrease in means when modifiers were present was again consistent with our predictions.

**Fig. 10:**
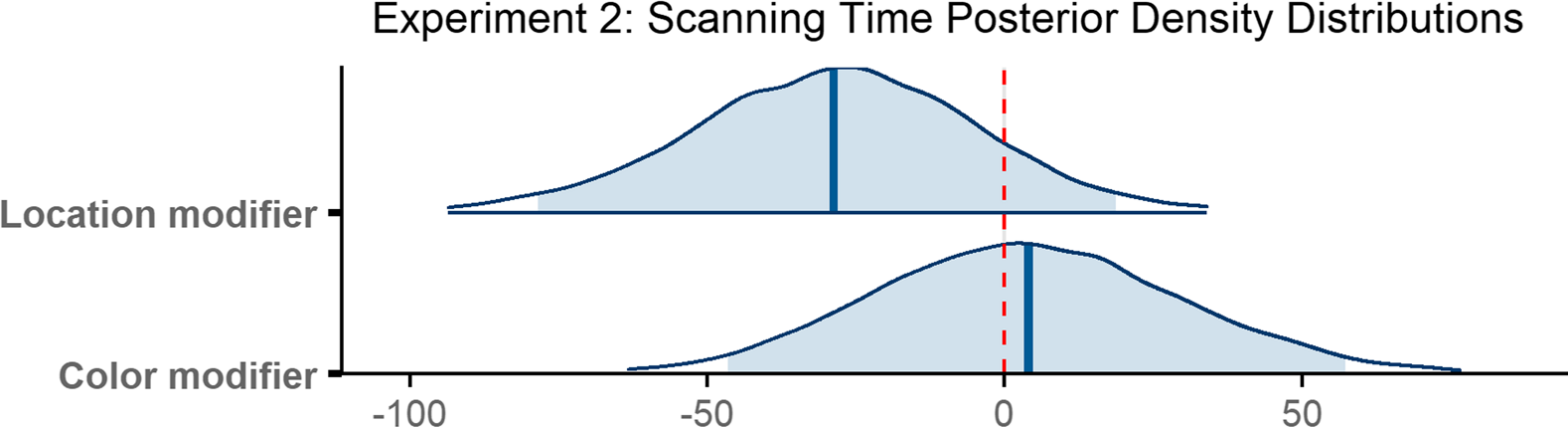
Posterior density distributions for scanning time *β* parameter estimates for each level of the fixed effect relative to the reference level. Light blue shaded regions indicate the 95% credible interval and blue vertical lines indicate the mean of the distribution. The red vertical dashed line highlights 0 on the *x* axis. If the red line falls within the light blue shaded region, the estimate is not reliably different from zero and interpreted as a null result

To analyze scanning time, we constructed a Bayesian mixed-effects model, which ran for 6000 total iterations. The model did not reveal a reliable difference in scanning time when there was a color modifier as compared to no modifier (*β* = 4.03, 95% CI = [− 46.48 57.36]). Scanning time also did not reliably differ when a location modifier was provided as compared to when no modifier was given (*β* = − 28.73, 95% CI = [− 78.48 18.68]; Fig. 10).

### Verification time

Verification time was 785 ms on average (*M* = 785 ms, SD = 784 ms). Verification required the most time when no modifier was present (*M* = 812 ms, SD = 781 ms), was shorter when a location modifier was present (*M* = 780 ms, SD = 775 ms), and was fastest when a color modifier was provided *(M* = 762 ms, SD = 796 ms; see Fig. 8c). Numerically speaking, the decrease in verification time when either type of modifier was present supported our prediction.

**Fig. 11:**
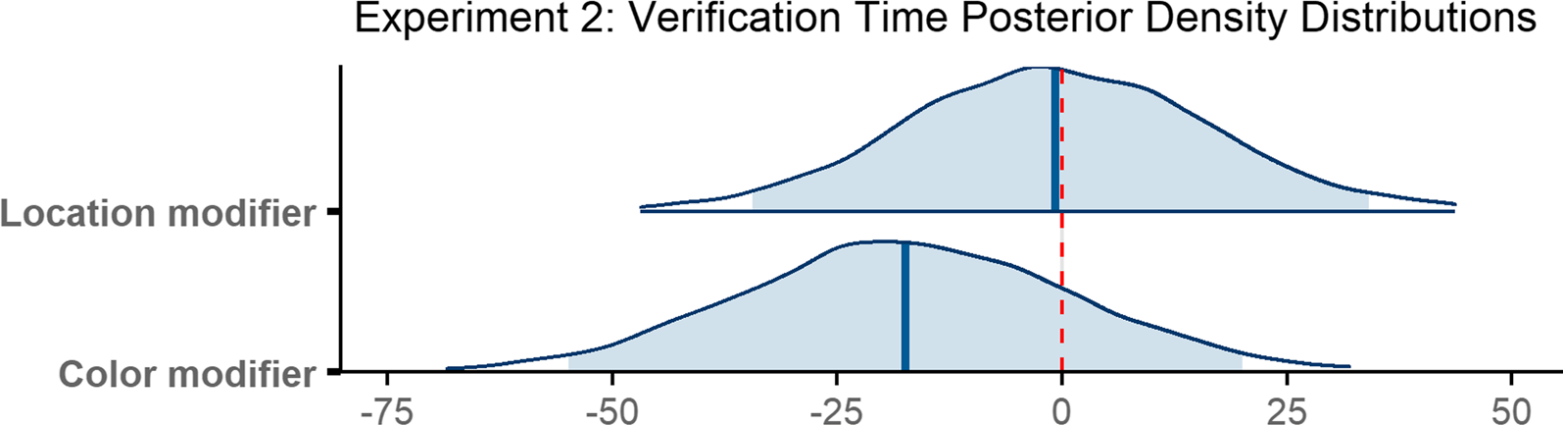
Posterior density distributions for verification time *β* parameter estimates for each level of the fixed effect relative to the reference level. Light blue shaded regions indicate the 95% credible interval and blue vertical lines indicate the mean of the distribution. The red vertical dashed line highlights 0 on the *x* axis. If the red line falls within the light blue shaded region, the estimate is not reliably different from zero and interpreted as a null result

The final model ran for 4000 iterations. The model revealed that none of the differences reported above were reliable. Verification time did not reliably differ when a color modifier was present compared to when no modifier was used (*β* = − 17.36, 95% CI = [− 54.90 20.04]), nor did it reliably differ with the presence of a location modifier (*β* = − 0.73, 95% CI = [− 34.45 34.07]; Fig. 11).

### Reaction time

On average, observers took 1755 ms to indicate that they had successfully found the target object (*M* = 1755 ms, SD = 1104 ms). Reaction time was longest when no modifier was present (*M* = 1805 ms, SD = 1115 ms), was slightly shorter when a color modifier was present (*M* = 1737 ms, SD = 1124 ms) and shortest when a location modifier was used (*M* = 1724 ms, SD = 1073 ms; Fig. 8d).

**Fig. 12:**
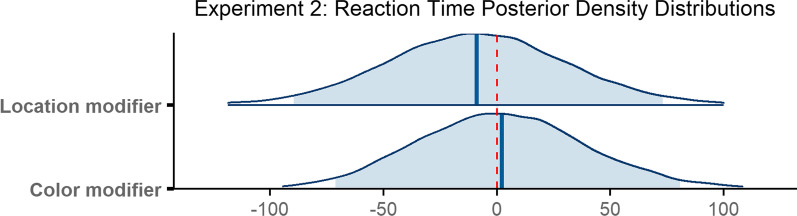
Posterior density distributions for reaction time *β* parameter estimates for each level of the fixed effect relative to the reference level. Light blue shaded regions indicate the 95% credible interval and blue vertical lines indicate the mean of the distribution. The red vertical dashed line highlights 0 on the *x* axis. If the red line falls within the light blue shaded region, the estimate is not reliably different from zero and interpreted as a null result

The final model ran for 6000 iterations. The model revealed that the small differences reported above were not reliable reaction time differences across conditions. Reaction time did not reliably differ when a color modifier was present compared to when no modifier was used (*β* = 2.22, 95% CI = [− 71.13 80.74]), or when a location modifier was present (*β* = − 8.86, 95% CI = [− 89.49 73.14]; Fig. 12).

In sum, the numerical trends for all measures were largely consistent with our prediction in that search was faster when a non-contrastive modifier was present as opposed to no modifier at all; however, unlike the results of Experiment 1, the numerical differences for the scanning epoch were not reliable (Fig. 12).

## Experiment 2: Discussion

In Experiment 2, non-contrastive modifiers did not enable observers to locate targets more quickly. Our results differed across Experiments 1 and 2 with respect to the primary search epoch: scanning time was faster in Experiment 1 for both modifier conditions relative to the unmodified baseline, but the presence of a modifier did not reliably facilitate scanning time in Experiment 2. A surprising aspect of these results is that the color modifier was the same in both experiments, yet only reliably facilitated search in Experiment 1. To further investigate the discrepancy across experiments, we conducted an exploratory analysis in which we compared search performance on the same measures analyzed previously, but only considered the scenes that were common to both experiments.

## Analysis of shared items

Results based on our primary measure of search performance (scanning time) differed across experiments. We may have expected the location modifier to have a different effect across the two experiments because it was not implemented the same way in both. However, we expected the color modifier to have the same effect in both experiments, but that is not what we found. To determine whether the differences can be attributed to the different scenes tested across experiments, we conducted an additional analysis on only the items and conditions that were common between both experiments for comparison.

There were 22 scenes shared across the two experiments. The dataset included only trials in the no modifier and color modifier conditions, as the search instruction was identical in these conditions across experiments. The combined dataset consisted of 621 trials from Experiment 1 and 636 trials from Experiment 2, a total of 1257 trials.

### Measures

Dependent variables in the current analysis included scanning time (our primary measure of interest) and reaction time.

### Analysis

The combined dataset was analyzed in the same manner as the individual datasets for each experiment, except that all Bayesian mixed-effects models included a main effect of experiment (1 or 2) and an interaction between experiment and modifier condition. The experiment fixed effect was centered prior to analysis. Additionally, because the intent of the analysis was to further examine the difference in facilitation from the color modifier across experiments, only the no modifier and color modifier conditions were included in the models. Each model included subject and item random effects with uncorrelated random slopes and intercepts, unless otherwise noted.

### Scanning time

**Fig. 13:**
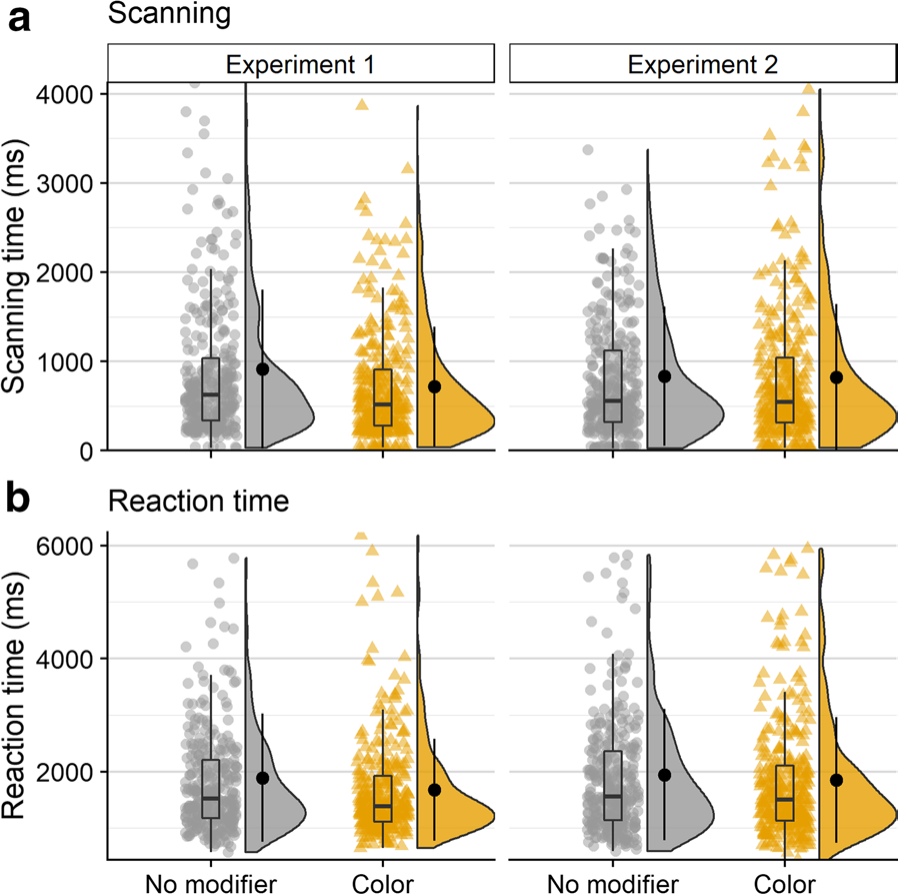
Raincloud plots depicting data for scenes shared across both experiments, oriented vertically for legibility. Points on the left side of each raincloud show raw data. Box plots, superimposed over the raw data, indicate the median value (middle horizontal line), the first and third quartiles (top and bottom lines in box), and 1.5 × the interquartile range: the distance between the first and third quartiles (whiskers). Violin plots show the range (minimum and maximum) and density (width) of the data. Isolated points on the right side of each raincloud indicate the mean, and error bars on said points reflect one standard deviation. For ease of visualization, the bottom 99% of the data are shown

Observers required 822 ms on average to scan the scene prior to fixating the target for the first time (*M* = 822 ms, SD = 798 ms). On average, scanning time was shorter in Experiment 1 (*M* = 816 ms, SD = 795 ms) than Experiment 2 (*M* = 827 ms, SD = 803 ms). When no modifier was present, scanning time was longer in Experiment 1 (*M* = 913 ms, SD = 892 ms) than in Experiment 2 (*M* = 834 ms, SD = 782 ms). When a color modifier was present, scanning time was shorter on average in Experiment 1 (*M* = 718 ms, SD = 670 ms) than in Experiment 2 (*M* = 820 ms, SD = 824 ms). The numerical trends were not consistent across experiments (Fig. 13).

**Fig. 14:**
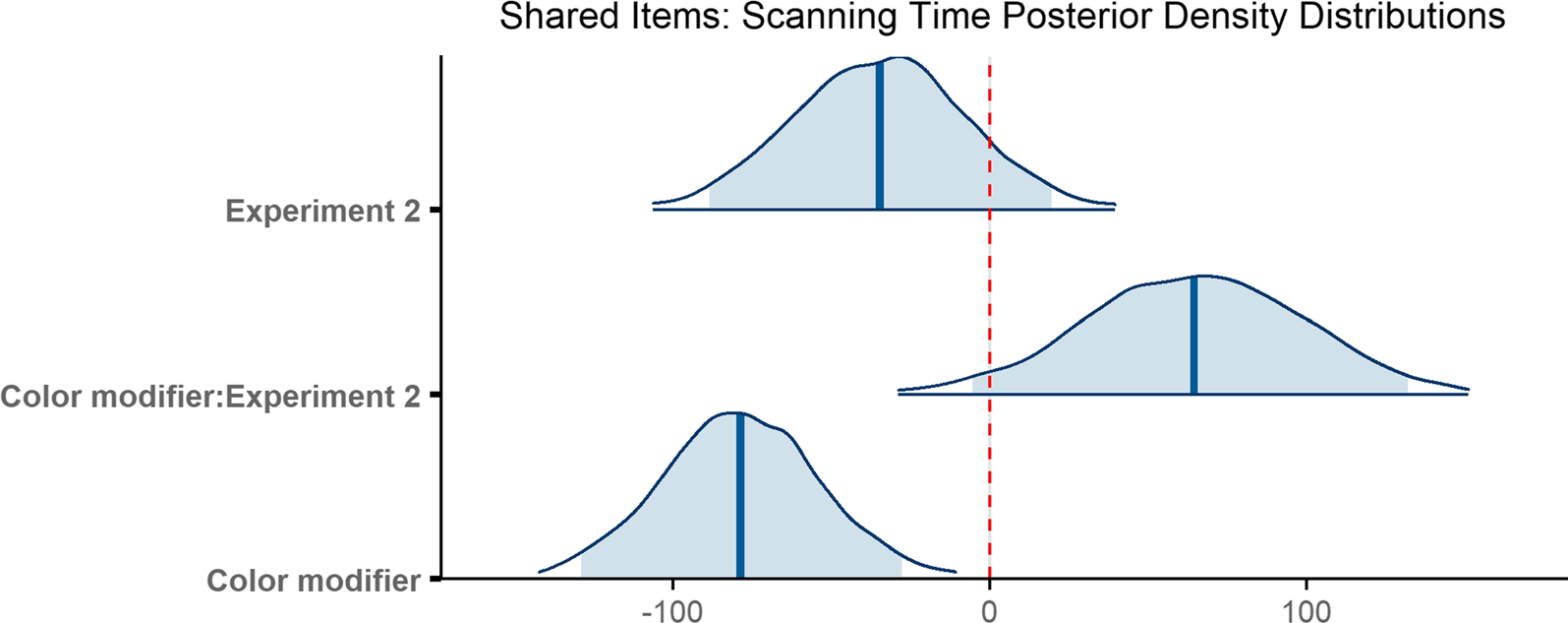
Posterior density distributions for scanning time *β* parameter estimates for each level of the fixed effect relative to the reference level. Light blue shaded regions indicate the 95% credible interval and blue vertical lines indicate the mean of the distribution. The red vertical dashed line highlights 0 on the *x* axis. If the red line falls within the light blue shaded region, the estimate is not reliably different from zero and interpreted as a null result

The final model ran for 4000 iterations. The model revealed that the difference in scanning time when there was a color modifier as compared to no modifier was reliable (*β* = − 78.64, 95% CI = [− 128.77 − 27.61]), and there was no simple main effect of experiment (*β* = − 34.64, 95% CI = [− 88.30 19.65]). With respect to the interaction between condition and experiment, slopes between the color modifier condition and the no modifier condition did not reliably differ across experiments (*β* = 64.43, 95% CI = [− 5.48 131.90]; Fig. 14). The results demonstrated an overall benefit of redundant color modifiers.

### Reaction time

Response times were 1842 ms on average (*M* = 1842 ms, SD = 1085 ms), and were shorter in Experiment 1 (*M* = 1784 ms, SD = 1028 ms) than Experiment 2 (*M* = 1898 ms, SD = 1136 ms). When no modifier was present, reaction times were shorter in Experiment 1 (*M* = 1891 ms, SD = 1132 ms) than in Experiment 2 (*M* = 1946 ms, SD = 1158 ms). Similarly, reaction times were shorter on average in Experiment 1 (*M* = 1677 ms, SD = 901 ms) than in Experiment 2 (*M* = 1850 ms, SD = 1114 ms) when a color modifier was present. The numerical trends were not consistent across experiments.

**Fig. 15:**
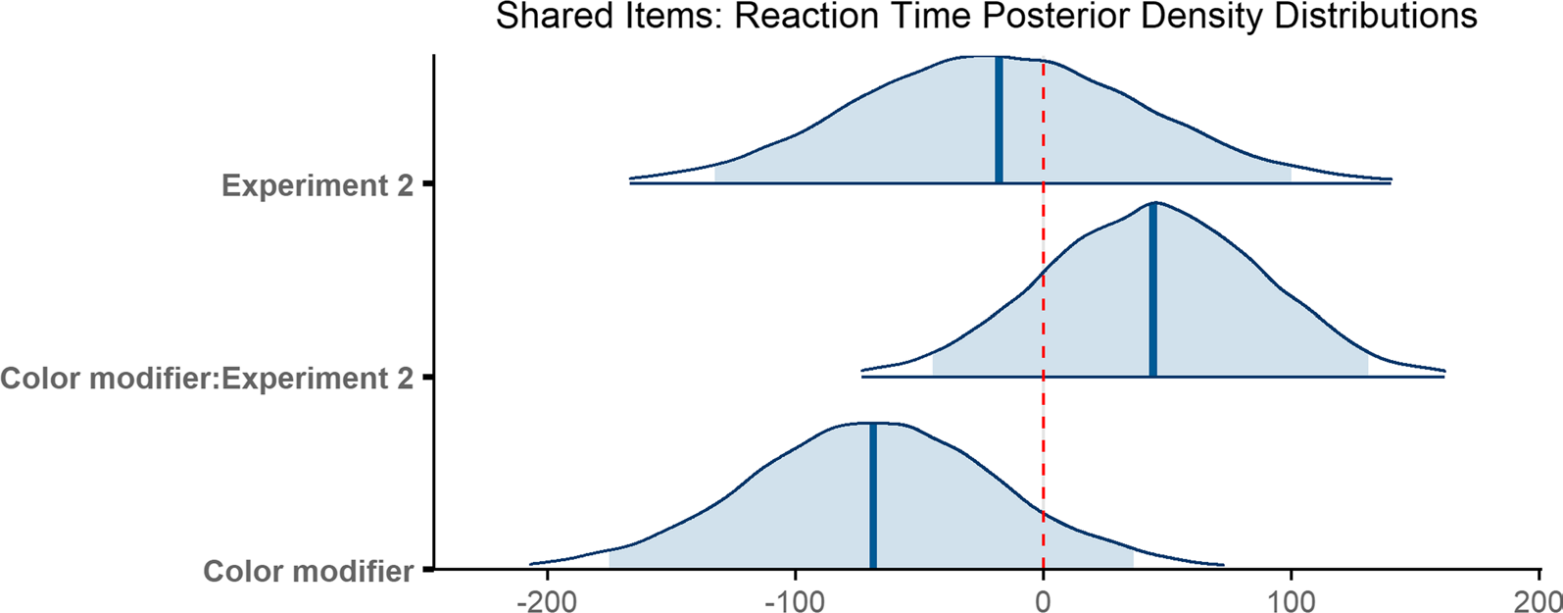
Posterior density distributions for reaction time *β* parameter estimates for each level of the fixed effect relative to the reference level. Light blue shaded regions indicate the 95% credible interval and blue vertical lines indicate the mean of the distribution. The red vertical dashed line highlights 0 on the *x* axis. If the red line falls within the light blue shaded region, the estimate is not reliably different from zero and interpreted as a null result

The final model ran for 8000 iterations total. The model did not converge with weakly informative priors, but did converge using more informative priors with a Student t distribution on β parameter estimates (*df* = 3, *μ* = 0, *σ* = 150) and on standard deviations for random effects (*df* = 3, *μ* = 0, *σ* = 150). The model revealed no reliable differences in reaction time when a color modifier was provided as opposed to no modifier at all (*β* = − 68.74, 95% CI = [− 175.13 36.40]). Similarly, the difference in reaction time across experiments was not reliable (*β* = − 17.89, 95% CI = [− 132.57 100.17]), nor did the slopes reliably differ for the color modifier vs. no modifier condition across experiments (*β* = 44.22, 95% CI = [− 44.61 131.16]; Fig. 15).

In sum, redundant color modifiers facilitated search overall during the scanning epoch, though the benefit of color modifiers was not observed for reaction time. We conclude that redundant color modifiers facilitated search for the scenes common to both experiments, and perhaps color modifiers were less useful in the new scenes that were introduced in Experiment 2.

## General discussion

The current study investigated whether perceptually relevant, but non-contrastive modifiers would facilitate visual search despite increasing the complexity of the search instruction. Specifically, we compared search duration when the search instruction included information about either the color of the target object or its location in the scene to performance when only the object name was given (e.g., *Find the lamp*). We predicted that non-contrastive color information would serve to augment the target object template and thereby facilitate template-guided search. We predicted that the addition of location information would facilitate search by constraining the region of the display to be searched.

Our findings for the primary search measure—scanning time—partially support our hypothesis that non-contrastive, but task-relevant modifiers improve reference precision (Degen et al. 2020) and therefore facilitate visual search. In Experiment 1, color modifiers facilitated search as measured by scanning time, likely by augmenting the target object template. Our findings for Experiment 1 are consistent with prior work on template-based guidance in visual search (Malcolm and Henderson 2009, 2010; Castelhano and Heaven 2010) and further demonstrate that color information can enrich the target template, consistent with Bahle et al. (2019), even when the color is communicated verbally. As predicted, the location modifier reduced search duration in Experiment 1 during the scanning epoch, consistent with Arts et al. (2011). The numerical trends in Experiment 2 were consistent with our predictions, the differences were not reliable. However, there was an overall benefit of color modifiers on scanning time as revealed by an analysis incorporating data from trials in both experiments that used the same scenes, suggesting that redundant color modifiers were generally beneficial.

In Experiment 1, the location modifier was more beneficial than the color modifier: While both modifiers helped observers locate the target faster, the benefit was larger for the location modifier. The simplest explanation for this observation is that location was more task-relevant. Template-based guidance, while certainly useful, is less useful in a search task than constraining the space to be searched. Because speakers frequently overmodify with color adjectives (Pechmann 1989; Belke and Meyer 2002; Sedivy 2003), it is also possible that color modifiers are less useful in part because they are more typical, or, at the very least, atypical modifiers may prime the observers to process the referring expression more carefully. Another possibility, consistent with Degen et al. (2020), is that color was a noisy, less-precise cue. A limitation of the current study is that we did not measure color entropy for the scenes tested (Toutouri et al. 2017). Augmenting the target object template with color information is less helpful in a scene with high entropy for that color (e.g., if multiple regions outside of the target object region share the target’s color). It is possible that color information did not improve reference precision if the target object’s color was well-represented in the scene. Future work should investigate whether a color modifier in the search instruction can facilitate visual search in real-world scenes better when color entropy is controlled for systematically.

The location modifier was clearly more beneficial in Experiment 1 than in Experiment 2. There are several possible explanations for why the modifier was more helpful in the first experiment. The first, and perhaps most obvious, is that the location modifier in Experiment 1 narrowed the region of the scene to be searched to a single quadrant (e.g., *on the top left*), which facilitated search because observers need not waste time exploring the other three scene quadrants. The instruction also allowed observers to rapidly orient to the region of the scene containing the target, as indicated by faster initiation times when the location modifier communicated the relevant scene quadrant to search. Indeed, observers may have decided where to search in the scene before the image appeared, as evidenced by faster reaction times for the location modifier in Experiment 1 only. The former explanation alone may be the reason for our disparate findings across experiments, but there are additional factors that may have rendered the modifier less useful in Experiment 2. The location modifier in Experiment 2 referenced an anchor object relative to the target object (e.g., *on the shelf*). For the anchor object information to be useful, observers first must extract scene gist and then use gist information to constrain the region of the display to be searched to where the anchor object would be likely to be in the scene. While scene gist extraction is rapid (Castelhano and Henderson 2007), unlike the quadrant information provided in Experiment 1, observers cannot benefit from gist before the scene appears. More importantly, the anchor objects varied in size, and in some cases referencing the anchor object may not have constrained the search region as much as in others, even if its location would have been highly predictable before the scene was shown (e.g., a shelf is probably in the upper part of the scene). In other words, the location information given in Experiment 2 was both noisier (anchor objects varied in size and predicted location), and less spatially constraining. Future work could address the latter limitation by controlling for the size of anchor objects in the scene.

At first glance, the difference between the results of Experiments 1 and 2 present a puzzle. In Experiment 1, the addition of non-contrastive but task-relevant modifiers clearly facilitated visual search, as evidenced by reliably faster scanning times. While the numerical trends fell in the predicted direction in Experiment 2, the differences were not reliable, suggesting that modifiers—even the color modifier, which was the same across both experiments—were far less beneficial, at least for scenes that were introduced in Experiment 2. It is possible that the new scenes were ill-suited to template-guided search using color information, perhaps due to differences in color entropy between the new and old scenes. Future work could address that possibility by measuring and controlling for color entropy in scenes. Another possibility is that the location modifier in Experiment 1, which constrained the search region to a single quadrant of the scene, was highly reliable, and the occasional inclusion of highly reliable information may have cued subjects to attend to and fully process the search instructions throughout the experiment. Given that the modifiers in Experiment 2 were noisier—or at least were less beneficial to subjects—it is possible that subjects used a shallow processing strategy when reading the search instruction (e.g., used good-enough processing, Ferreira 2003). Reading studies have shown that temporarily ambiguous sentences were read faster when they were followed by superficial questions about the sentences as opposed to when the questions probed how the ambiguity was interpreted (Swets et al. 2008; Tan and Foltz 2020). In other words, task difficulty modulated how carefully subjects read the sentences, because reading the sentence carefully would improve performance on difficult comprehension questions, suggesting that subjects use shallow, good-enough reading strategies when reading more carefully offers them no clear benefit. Crucially, the aforementioned task effect occurred even though subjects only encountered difficult comprehension questions 33% of the time. It is possible that observers in Experiment 1 read the search instruction more carefully than those in Experiment 2 because doing so made the task considerably easier for them when the instruction contained a location modifier (33% of the time). This may also explain why the scanning epoch was shorter in Experiment 2 on average than in Experiment 1: If observers came to rely on the information redundant modifiers provided in Experiment 1, search may have been slightly more difficult in their absence. By contrast, in Experiment 2 observers may have skimmed the instruction on each trial for the name of the target object (the head noun of the target phrase) while ignoring other information (any modifiers). It may be advantageous for observers to employ a shallow processing strategy when reading the search instruction if the redundant information is not especially useful and if maintaining the information in working memory would impose a working memory load. Future research could investigate whether good-enough processing of the search instruction predicts slower search by collecting reading measures on redundant modifiers in the search instruction and determining whether evidence for shallow processing (faster reading times or higher skip rates) for redundant information predicts slower search performance.

One surprising aspect of our results is that the search instruction affected initiation time in Experiment 1 only, which was not expected because no benefit of target template enrichment on search initiation time was reported in prior work (Malcolm and Henderson 2009, 2010). However, we suspect the difference is related to the nature of the location modifier in Experiment 1 rather than the effect of enriching reference precision more generally. The location modifier in Experiment 1 communicated which quadrant of the scene, specifically, contained the target object. It is therefore not surprising that subjects initiated search more rapidly when the instruction reduced the search region under consideration to a single quadrant, which allowed subjects to more rapidly select a region to foveate within that quadrant by ruling out candidate regions in other quadrants. An alternative explanation is that subjects shifted their gaze from the center of the screen to the aforementioned screen quadrant, and subsequently initiated search within the quadrant. The current study is unable to differentiate between these two possible explanations.

Consistent with previous paradigms used both in the visual search and reference processing literatures, our task used simple referential expressions (such as “Find the lamp”) as the search instruction, and made as few changes to the instruction as possible across conditions. The current study cannot speak to the naturalness of these simple referential expressions in everyday contexts, and it is possible that speakers would formulate search instructions differently. In a future study, the question could be addressed empirically by providing subjects with the scenes and target objects, then asking them either to formulate a search instruction de novo, or to complete a search instruction prompt (e.g., *Find the _____*). The consistency of the instructions produced for each scene could inform the results of the current study (e.g., perhaps the search instructions we used were more natural for some scenes than others). The productions could then serve as search instructions in a follow-up visual search task. Such a production task could also inform how speakers formulate referential expressions; namely, to determine how cooperative (or Gricean) speakers are when producing referential expressions for an interlocutor who will later search the image for the target: for example, perhaps speakers will produce a natural “*on the desk*” location modifier completion when there is no interlocutor (e.g., they are not told someone will use the instructions to search for the target) but would use a more helpful modifier like “*on the top left*” when that information could ostensibly be useful to another party.

It is also unclear how including both a color modifier and a location modifier in the search instruction might have influenced search behavior. On the one hand, adding linguistic material might bias observers to process the instructions in a good-enough manner, in which case observers should take longer to search scenes with multiple modifiers present than those with only one modifier; on the other hand, the cues may be additively beneficial (as in Malcolm and Henderson 2010; Castelhano and Heaven 2010; Hout and Goldinger 2015)—in which case having both a color and location modifier would result in faster scanning times than the location modifier alone—or performance when a color and location modifier are both present might be the same as when only an informative location modifier is present, which would suggest only the most informative cue dominates.

This study expands on work that has shown spoken language presented concurrent with scene viewing can facilitate visual search (Spivey et al. 2001; Tyler and Spivey 2001), even when the spoken information is redundant (Lupyan and Spivey 2010). We have shown that non-contrastive information presented in written form prior to scene viewing facilitates template-guided visual search, so long as the non-contrastive information is useful for referent identification.

Our results have practical implications outside of the laboratory. Interestingly, our findings suggest that neither of the two intuitive assumptions about the inclusion of non-contrastive modifiers is necessarily correct: That is, contrary to traditional Gricean accounts, speakers need not be as efficient as possible, or non-redundant in their communications, but contrary to some rational speaker accounts, inclusion of non-contrastive modifiers—even those relevant to target identification—is not always beneficial. Non-contrastive modifiers helped only when they added sufficiently useful information to the search instruction, which suggests that instructions in other domains (e.g., in the context of real-world navigation, in the design of a smartphone app or website, or in medical instructions) should be as clear, direct, and minimal as possible, and information beyond what is required for referent identification should only be added when that information is unambiguously beneficial to the user. Interestingly, our findings suggest that simply piling on modifiers to assist in the formation of a more precise template is not necessarily helpful, as the utility of one modifier (e.g., color) may depend on the reliability of any others and will vary depending on whether the modifier picks out a typical or atypical property. In other words, practical, real-world decisions concerning details to include in instructions should be made strategically. Instructions should be as simple and straightforward as possible, with non-contrastive modifiers provided if they are likely to facilitate performance given the other co-present sources of information.

## Conclusion

The current study investigated whether redundant but perceptually relevant information about the target object would facilitate visual search in real-world scenes. We conducted two eye-tracking visual search experiments in which we either included non-contrastive information in the search instruction about the color or location of the target in the scene, or provided only the name of the target object. Task-relevant, redundant modifiers in the search instruction facilitated visual search only when one such modifier was highly reliable. Consistent with Degen et al. (2020), we conclude that referring expressions containing non-contrastive information can nevertheless be appropriately informative—not overinformative—when the redundant information is useful, and that interlocutors engage in rational reference interpretation.

## Data Availability

All data and materials are available on the OSF: https://osf.io/uwqdm/.

## Footnotes

1 In supplemental analyses, we additionally examined spatial efficiency using scan path ratio and conducted shared item analyses on all of the dependent variables reported individually, for all experimental conditions. See supplemental materials on the OSF for details.
